# Autonomous Optimization of Targeted Stimulation of Neuronal Networks

**DOI:** 10.1371/journal.pcbi.1005054

**Published:** 2016-08-10

**Authors:** Sreedhar S. Kumar, Jan Wülfing, Samora Okujeni, Joschka Boedecker, Martin Riedmiller, Ulrich Egert

**Affiliations:** 1 Laboratory of Biomicrotechnology, IMTEK - Department of Microsystems Engineering, University of Freiburg, Freiburg, Germany; 2 Bernstein Center Freiburg, University of Freiburg, Freiburg, Germany; 3 Machine Learning Lab, Department of Computer Science, University of Freiburg, Freiburg, Germany; 4 BrainLinks-BrainTools Cluster of Excellence, University of Freiburg, Freiburg, Germany; Oxford University, UNITED KINGDOM

## Abstract

Driven by clinical needs and progress in neurotechnology, targeted interaction with neuronal networks is of increasing importance. Yet, the dynamics of interaction between intrinsic ongoing activity in neuronal networks and their response to stimulation is unknown. Nonetheless, electrical stimulation of the brain is increasingly explored as a therapeutic strategy and as a means to artificially inject information into neural circuits. Strategies using regular or event-triggered fixed stimuli discount the influence of ongoing neuronal activity on the stimulation outcome and are therefore not optimal to induce specific responses reliably. Yet, without suitable mechanistic models, it is hardly possible to optimize such interactions, in particular when desired response features are network-dependent and are initially unknown. In this proof-of-principle study, we present an experimental paradigm using reinforcement-learning (RL) to optimize stimulus settings autonomously and evaluate the learned control strategy using phenomenological models. We asked how to (1) capture the interaction of ongoing network activity, electrical stimulation and evoked responses in a quantifiable ‘state’ to formulate a well-posed control problem, (2) find the optimal state for stimulation, and (3) evaluate the quality of the solution found. Electrical stimulation of generic neuronal networks grown from rat cortical tissue *in vitro* evoked bursts of action potentials (responses). We show that the dynamic interplay of their magnitudes and the probability to be intercepted by spontaneous events defines a trade-off scenario with a network-specific unique optimal latency maximizing stimulus efficacy. An RL controller was set to find this optimum autonomously. Across networks, stimulation efficacy increased in 90% of the sessions after learning and learned latencies strongly agreed with those predicted from open-loop experiments. Our results show that autonomous techniques can exploit quantitative relationships underlying activity-response interaction in biological neuronal networks to choose optimal actions. Simple phenomenological models can be useful to validate the quality of the resulting controllers.

## Author Summary

Electrical stimulation of the brain is increasingly used to alleviate the symptoms of a range of neurological disorders and as a means to artificially inject information into neural circuits in neuroprosthetic applications. Machine learning has been proposed to find optimal stimulation settings autonomously. However, this approach is impeded by the complexity of the interaction between the stimulus and the activity of the network, which makes it difficult to test how good the result actually is. We used phenomenological models of the interaction between stimulus and spontaneous activity in a neuronal network to design a testable machine learning challenge and evaluate the quality of the solution found by the algorithm. In this task, the learning algorithm had to find a solution that balances competing interdependencies of ongoing neuronal activity with opposing effects on the efficacy of stimulation. We show that machine learning can successfully solve this task and that the solutions found are close to the optimal settings to maximize the efficacy of stimulation. Since the paradigm involves several typical problems found in other settings, such concepts could help to formalize machine learning problems in more complex biological networks and to test the quality of their performance.

## Introduction

Electrical stimulation of the brain is considered an effective strategy to manage the symptoms of an increasing range of neurological disorders like essential tremor [1, 2], dystonia [3] and Parkinson’s disease (PD) [4–7], and as a possible means to artificially inject information into neural circuits, e.g. towards neurotechnical prostheses with sensory feedback [8]. The response elicited, however, typically results from interaction of the stimulus with uncontrolled ongoing neuronal activity. The changes of neuronal activity induced by the stimulus are thus not only added to the continuing dynamics of neuronal activity but may be modulated by it. Under these circumstances, using constant stimulation would elicit very different responses in each trial [9, 10] and is therefore unsuitable to induce defined response features. To achieve stable responses, or to modulate them predictably, stimulation settings would need to adapt to the dynamics of the brain’s activity.

Without suitable models to capture the interaction between stimulus and ongoing activity and to characterize the underlying network mode, it is not possible to adjust stimulation such that a desired response is consistently achieved. Biologically mechanistic and analytically tractable models are, however, challenging to develop for a variety of reasons: Interactions may be non-linear, and may depend on network modes, on the dynamics of individual neurons and other factors [11–13]. Experiments in *in vivo* model systems show that the measured response of a network is strongly modulated by its state at the time of stimulation [14, 15]. Network states are, however, problematic to define explicitly because of the non-stationary nature of activity dynamics, uncertainty about the relevant spatial and temporal scales of influence and partial observability of the system. They are typically identified in retrospect. To illustrate this, consider UP and DOWN states observed in the neocortex [16–18] as network modes. The UP (resp. DOWN) state can be quite clearly identified based on intracellular recordings of the membrane potential and spike activity. Based on extracellular recordings of spikes alone, however, it would not be known during an inter-spike interval if the network had already transitioned to the DOWN (resp. UP) state at the time of stimulation. The momentary state of the network would be invisible, thus making it difficult to adjust stimulus settings online. Repetitive stimulation may even lead to interaction between responses. Furthermore, as studies *in vitro* suggest, this influence may affect responses across several scales of organization, from individual neurons [19] to networks [20, 21].

Despite the challenges, promising results from recent studies even with simple feedback strategies make a compelling case for closed-loop neurostimulation devices. Experiments on a primate model of PD indicated that even simple adaptive methods were superior to conventional open-loop Deep Brain Stimulation (DBS) [22]. Furthermore, the first report of event-driven DBS on human patients with PD [23] demonstrated an improvement in symptoms compared to standard DBS, with a simultaneous reduction in stimulation time. Such event-driven paradigms monitor a pre-determined indicator function in the spontaneous activity. In the most simple version, these indicators trigger fixed stimuli [22, 23] but do not modify the stimulus parameters as such. Where the quantitative input-output relations are known, predefined controllers could be successful. Keren and Marom [21], for example, achieved stable response probabilities with a PI controller to adjust the stimulus based on responses elicited by previous stimuli since, under the conditions selected, the input-output dependence was nearly linear.

Because of a lack of quantitative models that could be used to predict the ideal stimulus settings for the systems studied, the notion of optimality does not exist in these stimulation paradigms. Further, the nature of the problem in these examples was such that it was possible to define a singular target value a priori, i.e. to stop oscillatory activity [22] or to achieve a quantitatively predefined response feature, here, a fixed probability for a response [21]. When the quantitative value of the target response cannot be clearly defined, is intrinsically variable, or where multiple interacting objectives have to be balanced, e.g. a cost function exists, these approaches cannot be applied. To address such problems, we propose a reinforcement learning (RL) based closed-loop paradigm to autonomously choose optimal control policies without the need for a model of the system and its interaction with electrical stimulation.

The objective of this paper is to demonstrate in a proof-of-principle study how an RL based controller may be used to autonomously adjust stimulus settings to maximize stimulus efficacy in interaction with a generic network of neurons. The approach poses the following questions: (1) How to represent the interaction of network activity, stimulus and response as a quantifiable ‘state’ so that a well posed control problem may be formulated for variable conditions without predefining a singular target value, (2) how to find the optimal state for stimulation autonomously, (3) how to evaluate the quality of the solution found.

To develop the concept and techniques, we employed generic neuronal networks *in vitro* on substrate integrated microelectrode arrays as a model system. Previous studies offer a partial understanding of the dynamics in such networks and of the rules governing their interaction with electrical stimuli [20], which allowed us to test for the quality of the solutions found by an RL-based controller.

Neuronal networks in cell culture exhibit activity characterized by intermittent network-wide spontaneous bursts (SB), separated by periods of reduced activity. Electrical stimulation of the network also evokes bursts of action potentials (responses). Response strengths depend on the stimulus latency relative to the previous SB, and can be described by a saturating exponential model [20]. For this system we thus posed the following optimization problem: find the stimulus latency that would maximize the stimulation efficacy measured as the number of spikes evoked per SB in the face of ongoing network activity. The achievable efficacy is thus not known a priori and further properties of the network may be relevant to its value, i.e. dependencies may be incompletely captured in the model. According to [20], choosing longer latencies to stimulate ensure that longer responses are evoked, but are more prone to interruption by the next SB and thereby affects stimulation efficacy adversely by losing out on opportunities to stimulate. Choosing shorter latencies, on the other hand, ensures that stimuli are delivered more often without interruption by SBs, but at the cost of evoking weaker responses. To maximize stimulation efficacy in this context, we thus need to balance the trade-off between these opposing strategies. In this study, we asked if an RL based controller can autonomously find the ideal balance in this trade-off and identify the optimal stimulus latency.

The control problem thus formulated has several interesting features that make it pertinent to the problem at hand. To find the optimal time, a controller would have to reconcile the dynamic interplay of multiple biological processes, namely: a) the initiation of synchronous SBs in the network, b) recovery of the network excitability after SB termination, and c) the overall level of excitability of the network.

The controller has also to account for the modulation of system dynamics over a broad-range of time-scales. Furthermore, though these networks are similar w.r.t. statistical properties, every network has distinct dynamic features and unique connectivity. The controller thus needs to be able to operate robustly over a range of activity modes. Out of a high dimensional spatial and temporal feature space available in the recording, a relevant low dimensional quantitative state feature has to be abstracted and a strategy to converge toward optimal performance worked out. For these reasons, we argue that this toy problem captures many of the relevant challenges faced by closed-loop paradigms in biological frameworks and by RL based controllers in a complex adaptive environment. Finally, drawing on simple quantitative notions from previous studies, we computed network-specific optimal stimulation latencies [20] from open-loop data to independently validate the optimality of the learned controller. We observed that the learned stimulation latencies and achieved stimulation efficacies correlate strongly with the offline optimal values estimated for these networks.

Our results demonstrate the capacity of autonomous techniques to exploit underlying quantitative relationships in neurotechnical interaction with neuronal networks to choose optimal actions and illustrate how phenomenological models can be used to help formulate the RL problem and validate the performance of the resulting controllers.

## Materials and Methods

### Model system

The dynamics of neuronal activity *in vivo* is dependent on a multitude of factors including and not limited to cross-structural influences and specific anatomy and connectivity of the region of interest. Biological complexity in this scale makes it difficult to glean a consistent understanding of signal relationships between the network and an external stimulus, a crucial step in developing feedback control techniques [24]. In order to develop our concepts and algorithms, we used a model that while being generic and independent of specific functions and/or modalities preserves the biophysical complexity of the neuronal ensemble and relevant challenges an autonomous controller would face in a more complex context.

Neuronal networks grown on substrate integrated microelectrode arrays are a suitable model in that they are easily accessible generic neuronal networks that can be maintained in a controlled environment, exhibit spontaneous activity known to influence the network’s interaction with external stimuli, and are known to operate in distinct network modes across a wide-range of time scales. Furthermore controlling such biological neuronal networks poses many interesting challenges for research in RL such as potentially high dimensional state spaces, continuous action spaces and non-stationary dynamics.

Frontal cortical tissue was dissected from newborn Wistar rats (obtained from the breeding facilities of the University of Freiburg) after decapitation, enzymatically dissociated, and cultured on polyethyleneimine (Sigma-Aldrich, St. Louis, MO)-coated microelectrode arrays (MEAs; Multi Channel Systems, Reutlingen, Germany). The culture medium (1 ml) consisted of minimal essential medium supplemented with 5% heat-inactivated horse serum, 0.5 mM L-glutamine, and 20 mM glucose (all compounds from Gibco Invitrogen, Life Technologies, Grand Island, NY). Cultures were stored in a humidified atmosphere at 37°C and 5% CO_2_—95% air. Medium was partially replaced twice per week. Neuronal density after the first day *in vitro* (DIV) ranged between 1500 and 4000 neurons/mm^2^. The final density after 21 DIV settled at 1500–2000 neurons/mm^2^, independent of the initial density. At the time of recording, network size thus amounted to 5 − 6 × 10^5^ neurons. Animal treatment was according to the Freiburg University (Freiburg, Germany) and German guidelines on the use of animals in research. The protocol was approved by the Regierungspräsidium Freiburg and the BioMed Zentrum, University Clinic Freiburg (permit nos. X-12/08D and X-15/01H).

### Electrophysiology

Neuronal activity was recorded inside a dry incubator with MEAs with 59 titanium nitride (TiN) electrodes of 30 μm diameter and 500 μm pitch (rectangular 6x10 grid). One larger electrode served as reference. The primary signal was amplified (gain 1100, 1–3500 Hz) and sampled at 25 kHz/12 bit (MEA 1060-BC; Multi Channel Systems). Online spike detection was done with MEABench (version 1.1.4) [25] at six to eightfold root mean square noise level for spike threshold.

Such networks of dissociated neurons *in vitro* exhibit spontaneous activity characterized by intermittent network-wide synchronous bursts separated by periods of reduced activity. Inter-burst intervals (IBI) in these networks fit an approximate lognormal distribution. Stimulating the network also evokes bursts of action potentials (response). The length of these responses at a chosen recording electrode can be modulated by the latency of the stimuli relative to the SB at that channel. Their relationship was shown by [20] to fit a saturating exponential model.

### Trade-off problem

**Fig 1:**
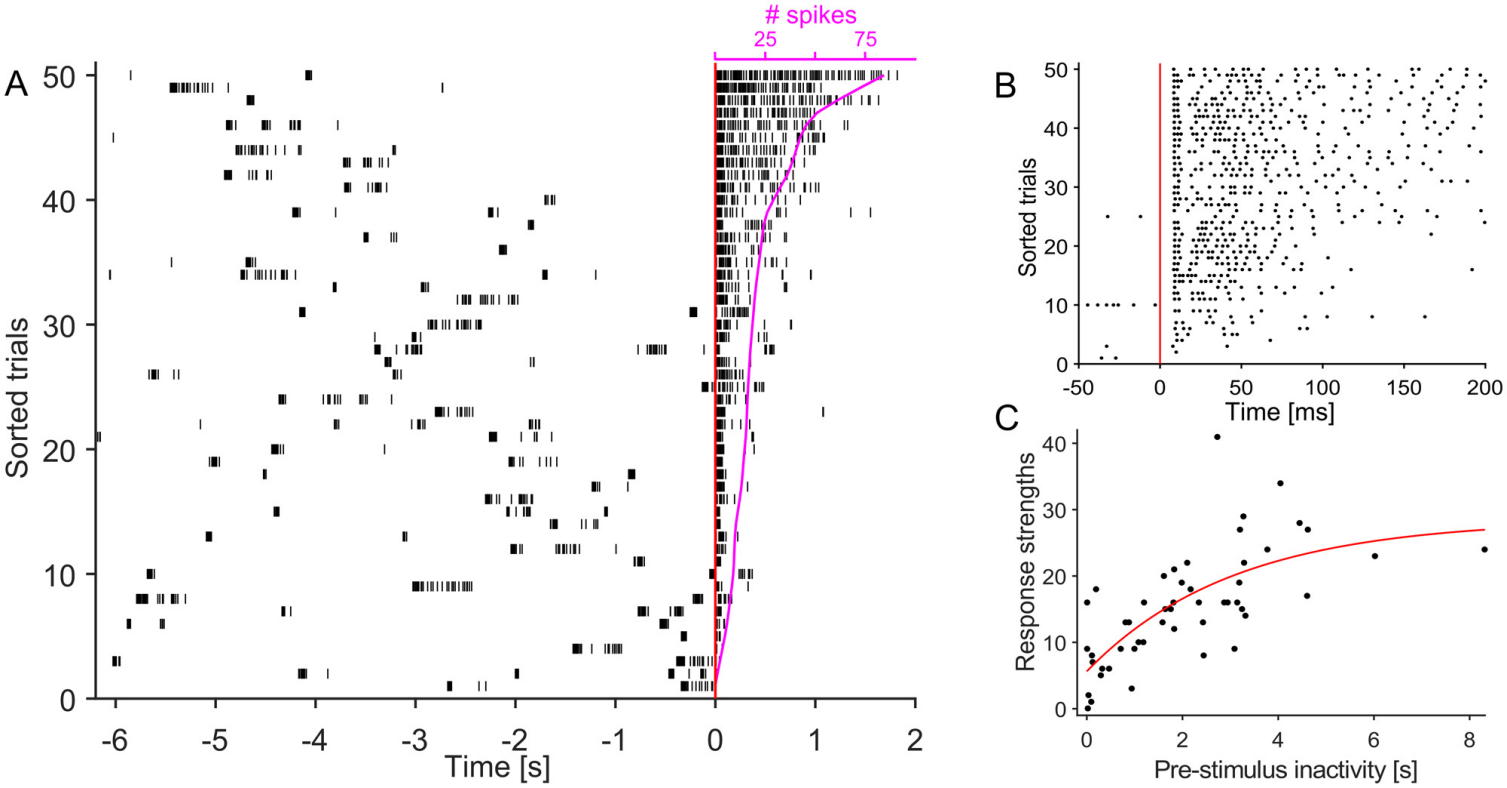
Stimulating the network at an electrode evokes a burst of activity. Response strengths were dependent on the period of inactivity preceding the stimulus. (A) Raster shows responses at one chosen recording channel in a network to 50 stimuli at the same electrode. Stimuli were delivered periodically, and thus at random latencies relative to the previous SB. Stimulation cycled through five pre-selected electrodes at 10 s intervals. Stimulus properties: -0.7 V, 0.4 ms, monophasic against common ground. Trials were aligned to the time of stimulation (red line) and sorted by the count of spikes within the designated response window (see magenta overlay). A response window of 2 s was chosen for this network. The diagram exposes the relationship of response strengths to the period of prior inactivity. The first 200 ms post-stimulus is zoomed in panel (B). Responses typically consisted of an early (≤ 20 ms post-stimulus) and late (≥ 50 ms post-stimulus) component. (C) The relationship between response strengths and periods of prior inactivity can be captured in a saturating exponential model similar to the dependency of response length [20].

The optimization problem was defined as the following: what is the optimal stimulus latency relative to the end of the previous SB at a selected recording electrode (RE) that would maximize the number of spikes evoked at that site per SB? To illustrate the problem, consider the following opposing strategies: A) choosing a long latency: Based on the saturating recovery model, longer latencies would elicit to longer responses (Fig 1). However, such a strategy would prove futile in the long run; long latencies are prone to interruptions by succeeding SBs and opportunities to stimulate will be forfeited. This would lower the count of evoked spikes per SB. B) choosing short latencies: this strategy would ensure that stimuli are delivered more often, but at the cost of evoking shorter responses. Optimization involves finding the trade-off between these opposing strategies. We asked that an RL based controller autonomously find the optimal time for stimulation to balance this trade-off for individual biological networks based only on the activity at the RE.

### Experimental procedure

Experiments were performed on 20 networks between day *in vitro* (DIV) 19 and 35 (‘network’ denotes a culture at a specific point in time). Each experiment began with one hour of recording spontaneous activity, from which bursts were detected offline. A statistical model of SB occurrence was estimated by fitting a lognormal function to the IBI distribution to extract the location and scale parameters (*μ* and *σ* respectively).

#### Selection of stimulation and evaluation ssites

Spontaneous data were further analyzed to identify MEA electrodes that would serve as sites of stimulation and of evaluation of the responses. As candidate stimulation electrodes (SE) we selected sites that were more likely to participate early in SBs [20]. This procedure identified the so-called “major burst leaders” [26, 27]. For open-loop stimulation, monophasic, negative voltage pulses of 400 μs width and 0.7 V amplitude were delivered at candidate SEs at 0.1 Hz. Final SE and RE pairs were typically selected based on peri-stimulus time histograms (PSTH) from positions with responses consisting of both early (≤ 25 ms) and late (≥ 50 ms) components.

#### Response strength

Following the choice of SE and RE we identified the dependence of response strengths on the periods of inactivity preceding stimuli for a given network. The number of spikes at the recording channel in a 500 ms window following a stimulus was typically defined as the response strength (RS). This data was used to estimate the parameters *A*, *B* and *λ* of the recovery model by least square fitting.

#### Closed-loop stimulation

**Fig 2:**
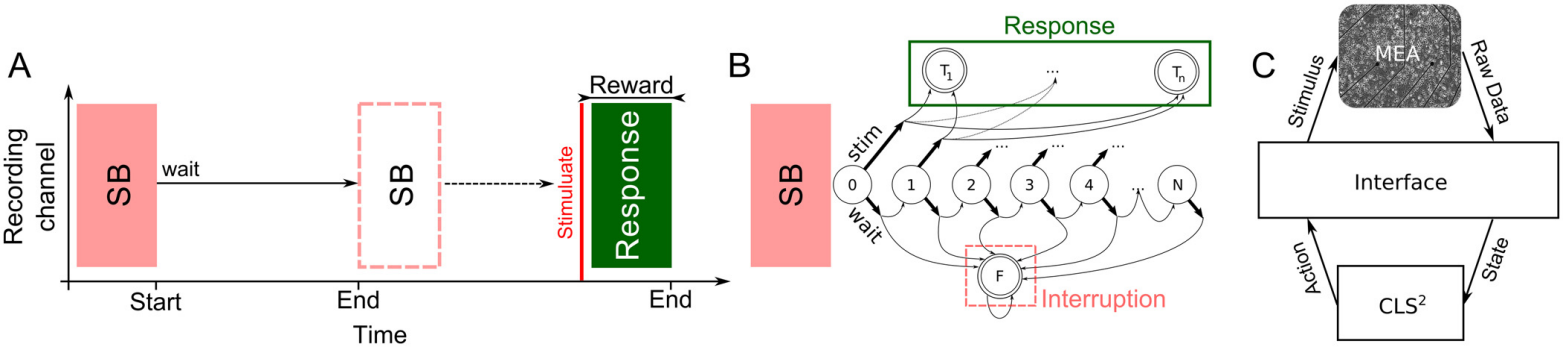
Stimulation trials and the closed-loop architecture. (A) A trial started with the end of a spontaneous burst (SB). The trial was terminated either by the next SB (dotted box) or a stimulation. In our paradigm, reward was defined as the number of spikes in the response. Interruptions by SBs led to neutral rewards (punishment). (B) The time within each trial was discretized into 0.5 s steps, corresponding to states 1, …, *N*. At each state, the controller could choose between two actions: to wait or to stimulate. A ‘stimulate’ action led to one of the terminal states *T*_*i*_, with *i* indicating the strength of the response. Terminal state *F* was reached if the trial was interrupted by ongoing activity. (C) Schematic visualization of the closed-loop architecture.

Closed-loop episodic learning sessions were performed using RE and SE positions identified as above. The controller was designed to learn in episodes that commenced at the termination of each SB (Fig 2A and 2B). The closed-loop architecture was realized by interfacing the data acquisition software (MEABench) with the closed-loop control software, CLS^2^ (Closed-loop Simulation System, Fig 2C). Learning sessions proceeded in alternating training and testing rounds. During training, the controller was free to explore the state-action space and learn a control law using the RL algorithm described in the following section, while during testing it always behaved optimally based on the knowledge hitherto acquired. Subsequent to the closed-loop session, spontaneous activity was recorded for one hour to check for non-stationarity in the IBI distribution. The stimulus properties were as during open-loop stimulation.

### Reinforcement learning

Learning a controller for a given task with RL requires formalizing it as a Markov Decision Process (MDP). An MDP is defined as a five-tuple (S, A, T, *R*, *P*), where S is a set of states, A a set of actions and T a finite set of discrete time points (finite horizon). The reward function R:S×A×S→R defines the reward an RL controller receives when it applies action $a_{t}\in A$ in state $s_{t}\in S$ and transitions into $s_{t+1}\in S$ at time t∈T. The probabilistic transition model *P* (*s_t+1_ | s_t_*, *a_t_*) defines the conditional probability of transitioning from state *s*_*t*_ to state *s*_*t*+1_ under the action *a*_*t*_. The goal of RL is now to find a control law (policy) π:S→A which maximizes the expected accumulated reward $V^{\pi }\left(s\right)=E\left\{\sum_{t=0}^{T}\gamma^{t}R\left(s_{t},\pi \left(s_{t}\right),s_{t+1}\right)|s_{0}=s\right\}$ where *γ* is a discounting factor on future rewards. Value Iteration is commonly used to find *V* if the transition model *P* is available. Although in this proof-of-concept study a model is available and used to verify the solution found by the RL controller a-posteriori, for biological systems in general we can not assume that a model is known. We therefore consider a model-free setting and use Q-learning [28] to learn an action-value function *Q*(*s*, *a*) (Q:S×A→R) which represents the value of choosing action *a* in state *s*. The greedy policy *π* can then be derived as *π*(*s*) = arg max_*a*_ *Q*(*s*, *a*). To apply Q-learning we first have to define the state and action space as well as a suitable reward function.

#### State space definition

Our definition of S is motivated by the following considerations. Solving the trade-off problem involves reconciling the dynamic interplay of the initiation of synchronous SBs in the network and the recovery of network excitability after SB termination. A simple statistical model of the initiation of synchronous SBs is a lognormal function of the period of inactivity between SBs. The cumulative of this distribution indicates the probability of SB initiation as a function of time after the preceding SB (Eq 4). At the same time, recovery can equally be modeled by an exponential function of the time after the end of an SB (Eq 5). Stimulation at a certain latency thus effectively probes the level of recovery at that time. This latency was defined as the quantitative state variable accessible to the learned controller, providing information on the dynamics of both processes. Therefore the time after SB termination is a simple and intuitive choice of a low dimensional state feature. We discretized this latency in 0.5 s steps, corresponding to states 1, …, *N*. These make up the set of states S together with terminal states that reflect the outcome of the stimulation *T*_*i*_ (*i* indicating the response strength) or an “interruption” state *F*.

#### Reward function

In order to learn the optimal stimulus latency, the controller needs to be appropriately rewarded/punished (Eq 1). In Fig 2B, within an episode, at each state the controller could choose between two actions: to ‘wait’ or to ‘stimulate’ which make up the action set A. An episode terminated either when a preset maximum number of states (i.e. maximal latency) was reached, an SB occurred or when a ‘stimulate’ action was chosen. After each episode, the controller received a terminal reward proportional to the strength of the evoked response. Alternatively, if an SB had occurred or the maximum number of cycles was reached it received a neutral reward (punishment):

$$R\left(s_{t},a_{t},s_{t+1}\right)=\left\{\begin{array}{cc} i, & \;\text{if}\;s_{t+1}=T_{i},i\in \left\{1,\ldots ,n\right\}\\ 0, & \text{otherwise}. \end{array}\right.$$ (1)

#### Q-Learning

As a learning algorithm, we used online Q-learning with a tabular representation of the Q-function. To guarantee full exploration of the state and action space the controller follows a random policy *π*_explore_ during training that uniformly chooses the state of stimulation. The Q-function is iteratively updated during training sessions as:

$$Q_{t+1}\left(s_{t},a_{t}\right)=Q_{t}\left(s_{t},a_{t}\right)+\alpha \left[R\left(s_{t},a_{t},s_{t+1}\right)+\gamma \max_{a}Q_{t}\left(s_{t+1},a\right)-Q_{t}\left(s_{t},a_{t}\right)\right],$$

where we set the learning rate to *α* = 0.5 and use no discounting (*γ* = 1) since we consider a finite horizon problem. During testing sessions the controller follows a greedy policy (Eq 2) without exploration:

$$\pi \left(s\right)=\underset{a}{arg max}Q\left(s,a\right)$$ (2)

### Data analysis

Offline burst detection was performed for spontaneous data using the following algorithm: For spikes recorded from each electrode: a) interspike interval (ISI) had to be ≤ 100 ms, b) an interval ≤200 ms was allowed at the end of a burst and defined the minimal IBI, and c) the minimum number of spikes in a burst was set to three. Furthermore, at least three recording sites had to have burst onsets within 100 ms, and only one larger onset interval ≤200 ms was allowed [20].

For online burst detection at a single chosen channel, an individual ISI threshold was defined for each network based on spontaneous activity at the channel of interest prior to the closed-loop session. The ISI distribution of spontaneous activity was typically bimodal, with a strong first peak corresponding to ISI within SBs and a second peak for the intervals between bursts. The minimum between the intra- and inter-burst intervals was chosen as the threshold. The minimum number of spikes in a burst was set to three.

Parameters extracted from the fitting procedures were used to compute t*, the open-loop parametric estimate of the optimal latency (Eqs 3–7). To compare the predicted and realized improvement in stimulation efficacy after learning we estimated the stimulation efficacy of a strategy using random stimulation latencies taken from the objective function as the baseline model. The efficacy of this strategy corresponds to the mean of the objective function of each network.

## Results

To analyze the performance of closed-loop autonomous control systems for neurotechnical interaction with neuronal networks we designed a reduced model system that captures several general aspects of the problem setting. Networks of cortical neurons in culture develop robust spontaneous activity that influence the outcome of stimulation in non-trivial ways. In addition, this activity exhibits non-stationarities that any control system needs to cope with. We further defined a target function whose quantity was not known to the control system a priori but needed to be identified autonomously. State and action space were restricted to keep the dimensionality of the paradigm low and allow quantitative validation of the optimization problem.

### Properties of spontaneous network activity and response to electrical stimulation

**Fig 3:**
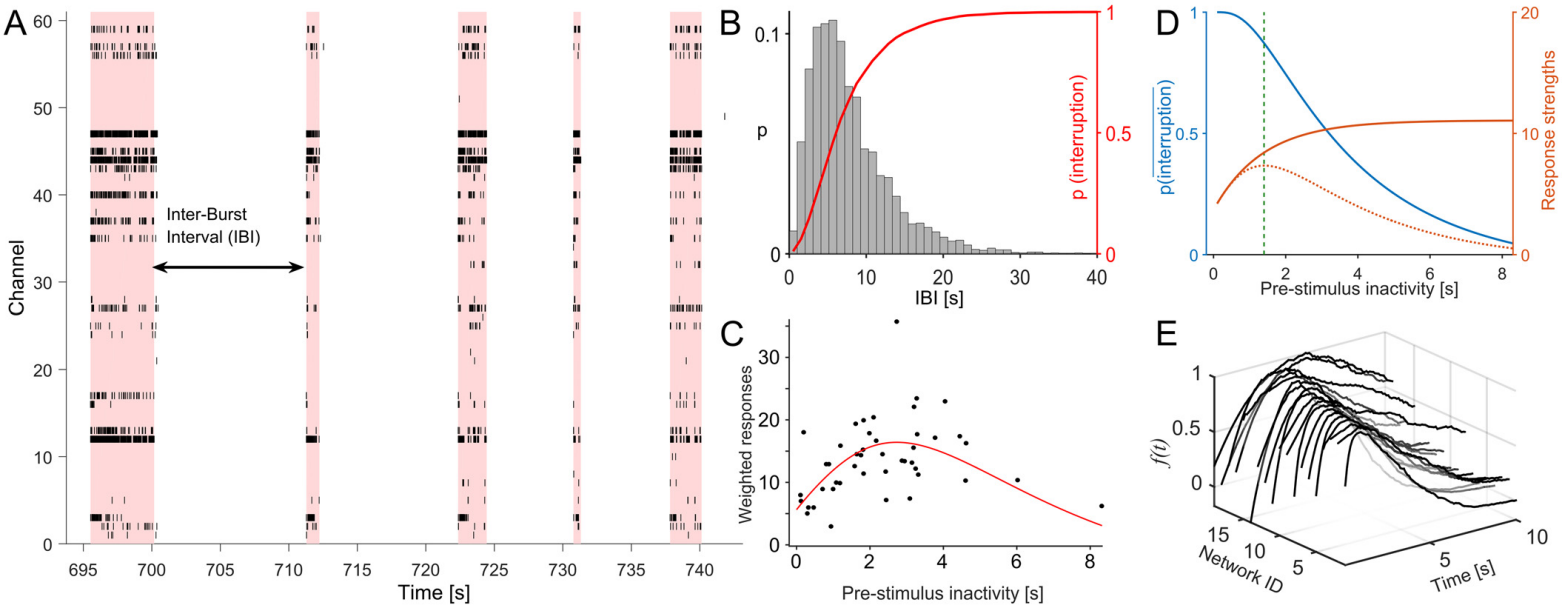
Identification of network specific objective functions. (A) Networks of dissociated neurons *in vitro* exhibit activity characterized by intermittent network-wide spontaneous bursts (SB) separated by periods of reduced activity (raster plot for 60 channels in a DIV 27 network). The shading marks the limits of individual SBs as detected by the burst-detection algorithm. (B) The distribution of Inter-Burst Intervals (IBIs) is approximately lognormal. The histogram shows the IBI distribution for the network in (A). The cumulative of this distribution (red) is predictive of the probability of being interrupted by ongoing activity given the elapsed period of inactivity, i.e. the current state *s*_*t*_. (C) Such a distribution was used to weight response strengths so that each dot represents the mean response strengths that can be evoked over a set of trials, including those that did not lead to stimulation, for a given stimulation latency. The fit predicts the objective function of the optimization problem. The example shows the data for the network shown in Fig 1C. The curve reveals a quasiconcave dependency, a unique global maximum and an optimal latency of ≈ 2.5 s in this network. (D) Fits to the probability of avoiding an interruption (blue), response strengths prediction (orange), and the resulting weighted response curve (orange, dotted) shown for another network. An optimal latency of ≈ 1.5 s emerges in this case. (E) All predicted objective functions for each of the 20 networks studied were quasiconcave and unique choices of optimal stimulus latencies were available. The objective functions were normalized to peak magnitude.

Neuronal networks cultured on MEAs display spontaneous activity that consists of synchronized network-wide spontaneous bursts (SB) separated by periods of inactivity. Burst-lengths ranged between hundreds of milliseconds to few seconds. SBs were detected using an algorithm that combined an inter-spike-interval threshold and the number of simultaneously active sites (Fig 3A). Inter-burst-intervals (IBIs) were approximately lognormal distributed (Fig 3B). Fitting algorithms yielded the location and scale parameters (*μ* and *σ*) of the corresponding lognormal distribution. The cumulative of this distribution was used to estimate the probability of another SB occurring given the period of inactivity that elapsed—or what we term the ‘probability of interruption’ following an SB (Fig 3B, red line).

Stimulating a network at a channel evoked a burst of activity at others. For our experiments, we selected one stimulating and recording channel each. Weihberger et al. [20] showed that the greater the duration of network inactivity, the longer the responses at a chosen site will be, according to a saturating exponential model. In order to verify this relationship and extract the parameters of the corresponding model, stimuli were delivered at random latencies relative to the previous SB (open-loop stimulation). Fig 1A shows responses at the recording channel to 50 such trials in an example network. Responses typically consisted of an early (≤20 ms post-stimulus) and late (>50 ms post-stimulus) component. The early component, presumably reflecting responses to antidromic stimulation, was characterized by temporally precise and reliable responses while the late component, presumably reflecting responses to orthodromic, transsynaptic activation, was both variable and unreliable (Fig 1B).

A least square fit of the response strengths to a saturating exponential model with stimulus latency as the independent variable was carried out. The fitting function was of the form *A*(1 − *e*^−*λt*^) + *B* (in red in Fig 1C). We then weighted all response strengths with the probability of being able to deliver a stimulus at the corresponding latencies, without being interrupted by ongoing activity. The weighted response strength curve (objective function) thus provides an estimate of the average number of response spikes that can be evoked for each SB (Fig 3C and 3D). A solution that maximizes this estimate is therefore the optimal solution to the proposed trade-off problem, namely, to find the stimulus latency that maximizes the number of response spikes per SB.

We observed that a unique optimal stimulus latency exists for each of the 20 networks we studied (Fig 3E). The optimal latency emerges as the result of interaction of processes underlying ongoing and stimulus evoked activity dynamics of the network. Quantitative insights from previous studies [20] allowed us to extract relevant parameters from recorded data. We then constructed a simple parametric model to compute the network-specific optimal latency offline, before we let the RL controller explore the problem in a closed-loop.

### Dependency of optimal stimulus latencies on properties of network activity

To understand the emergence of the optimal stimulus latencies from interacting biological processes and visualize the nature of the input–output relations and their relationship with the underlying parameter space, we considered simplified phenomenological models of each of the major contributing processes. Input, in the context of this problem refers to the period of inactivity/latency after which a stimulus is delivered, and output—the average number of response spikes evoked for every SB—the response feature of interest. The recovery of post-burst network excitability was modeled as a saturating exponential function (Eq 3). A statistical model of the temporal occurrence of SB events was considered (Eq 4). The corresponding model parameters were extracted from spontaneous and evoked activity recorded from each network.The model equations were:

$$R(t)\;=\;A(1-e^{-\lambda \;t})+B,$$ (3)

$$IBI(t)\;=\;\frac{1}{t\sigma \sqrt{2\pi }}e^{-\frac{{\left(ln\;t-\mu \right)}^{2}}{2\sigma^{2}}},$$ (4)

$$\bar{I}(t)\;=\;1-\Phi \left(\frac{ln\;t-\mu }{\sigma }\right),$$ (5)

$$\text{where }\Phi (x)\;=\;\frac{1}{\sqrt{2}\pi }\int_{-\infty }^{x}e^{-\frac{t^{2}}{2}}dt,$$

$$f(t)\;=\;\bar{I}(t)\cdot R(t),$$ (6)

$$t^{*}\;=\;\text{arg}\underset{t}{\text{max}}f(t).$$ (7)

*R*(*t*) and *IBI*(*t*) are the response strengths and the IBI respectively, modeled as a function of the period of inactivity, *t* (input). $\bar{I}\left(t\right)$ is the computed probability of avoiding an interruption, given a period of inactivity, *t*, and *f*(*t*) the appropriately weighted response strength model—the objective function (the input–output relationship). *f*(*t*)|*t* then gives the stimulus efficacy for repeated stimulation at latency *t*. The optimal latency t* is the maximizer of this function.

**Fig 4:**
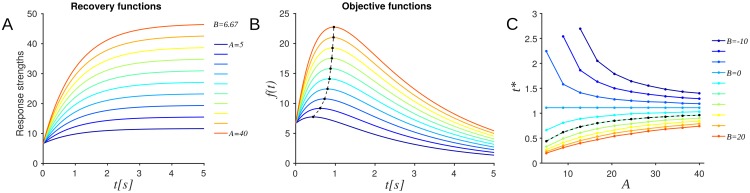
Dependence of optimal latency on parameters that capture the network’s response to stimuli. Dependence of the objective function on parameters that capture the network’s response to stimuli. In all panels the parameters *λ*, *μ* and *σ* were set to 6.67, 1, 0.6 and 1, respectively. (A) Changes of response strength with the gain *A* of the response strength model within the range observed experimentally (5 ≤ *A* ≤ 40, *B* = 6.67; *t*: stimulus latency) (B) The optimal latencies t* (dots), i.e. the maxima of the objective function *f*(*t*) increased non-linearly with the gain parameter *A* (dashed line). Color code as in panel A (*B* = 6.67). (C) Changes of optimal timing t* as a function of gain *A* and y-intercept *B* within the range observed experimentally (-10 ≤ *B* ≤20). *B* influences the relationship of t* with *A* and was trivial at *B* = 0. Black dots and dashed line indicate the case *B* = 6.67 shown in panel B. Note that *A* + *B* > 0 was imposed to ensure that the maximal responses were strictly positive.

In order to visualize the dependence of the input-output relations on the contributing parameters, we numerically computed objective functions and the corresponding t*, while varying one or more parameters and holding the remaining constant. Initially, *A* was allowed to vary while parameters *B*, *λ*, *μ*, *σ* were held constant. Fig 4A and 4B shows the family of recovery functions considered and the corresponding family of objective functions. In general, all objective functions shared the property of being quasiconcave and permitted a unique maximum. These maxima (marked as dots) were the desired outputs and the corresponding stimulus latencies t*, the desired optimal latency. The desired output– or equivalently the desired latency– increased non-linearly with *A* (Fig 4B).

**Fig 5:**
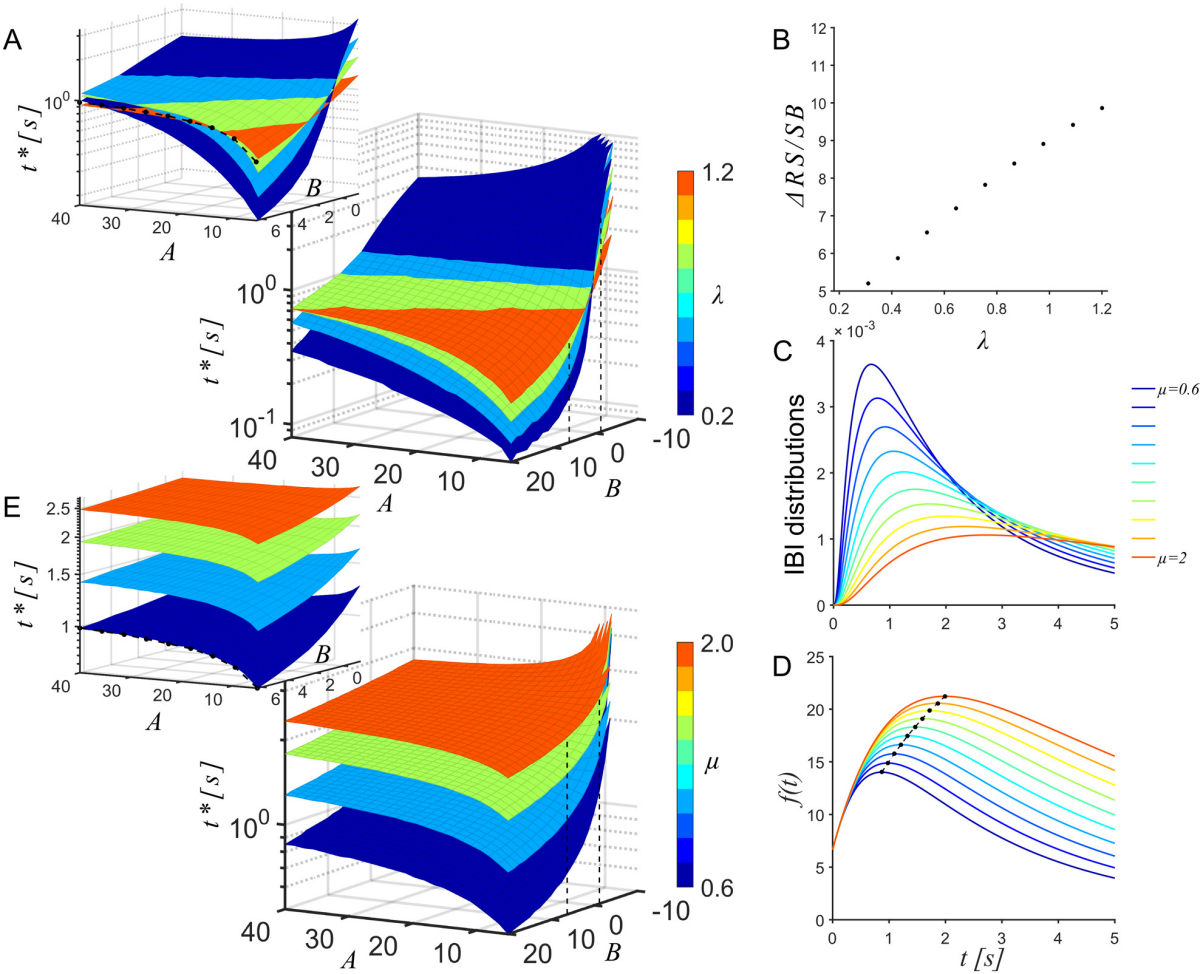
Dependence of the optimal latency on properties of the network’s activity dynamics. (A) Dependence of the optimal stimulus latency t* on the *A* − *B* plane. Each plane corresponds to a different value of the time constant *λ* of the recovery function within the range observed experimentally (0.2 ≤ *λ* ≤ 1.2). (inset) Zoom-in to −2 ≤ *B* ≤ 6.67 to reveal the monotonic rise of t* (dots and dashed line) that corresponds to the case described in Fig 4B (*λ* = 1). (B) Dependence of the gain in stimulation efficacy by using t* over random stimulation latencies on the time constant *λ* of the recovery function. *μ*, *A*, *B*, and *σ* were set to 0.6, 20, 6.67, and 1 respectively. (C) IBI distributions for the range of values observed experimentally of the location parameter *μ* (0.6 ≤ *μ* ≤ 2) for *A*, *B*, *λ*, *σ* set to 20, 6.67, 1 and 1 respectively. (D) The family of objective functions corresponding to the IBI distributions in (C) shows the near linear relationship of the optimal latencies with *μ* (dots and dashed line) (*A*, *B*, *λ*, *σ* were 20, 6.67, 1 and 1 respectively; colors as in (C)). (E) Summary of the dependence of the optimal stimulus latency on the *A*–*B*–*μ* space for *λ* = 1. Each plane corresponds to a different value of the location parameter *μ* of the IBI distribution. (inset) Zoom-in to −2 ≤ *B* ≤ 6.67) to reveal the rise of t* (dots and dashed line) that corresponds to the case described in Fig 4B (*λ* = 1, *μ* = 0.6).

Within the parameter range observed for *A* (mean and standard deviation 15.5 ± 9.3) and *B* (mean and standard deviation 4 ± 5.8) in our networks, the nature of the objective function family was preserved; a unique optimal latency existed, and monotonically increased or decreased non-linearly with *A*, depending on the value of *B* (Fig 4C). Fig 5A, summarizes the dependence of t* on the *A* − *B* plane. Each color coded plane corresponds to a different value of the time constant *λ*. *λ* was allowed to vary in the range observed experimentally (0.2 ≤ *λ* ≤ 1.2).

Next, we varied the location and scale parameters, *μ* and *σ* respectively—see Eq 4 of the IBI distribution. The corresponding input–output relations were still quasiconcave, thus ensuring the existence of a unique maximum. The optimal latency depended almost linearly on *μ* (Fig 5D). Fig 5E illustrates how the optimal latency is modulated in the *A* − *B* − *μ* space for *λ* = 1. The scale parameter *σ*, however, had no significant effects on the shapes of the objective functions and hence the corresponding optimal times (S1B Fig). The model thus allowed us to predict the optimal stimulus latency based on the individual properties of spontaneous and evoked activity of each network.

### RL based strategy to learn optimal latencies

**Fig 6:**
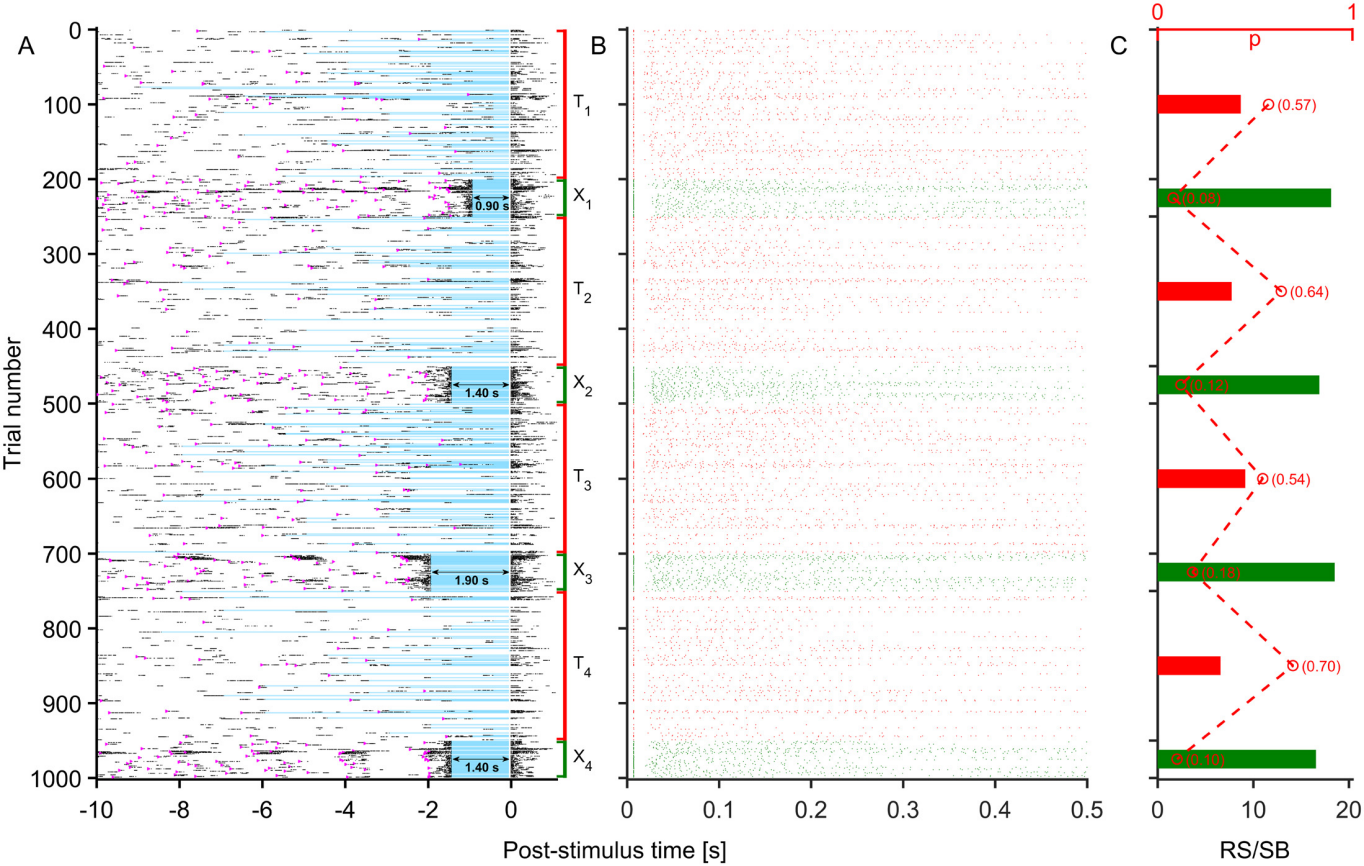
A closed-loop learning session in an example network. A closed-loop learning session in an example network. The session consisted of 1000 trials (200 training (T_i_, red), 50 testing (X_i_, green) trials and 4 such pairs) (A) Raster diagram showing the activity at the recording channel around the time of stimulation. Trials interrupted by ongoing activity are left empty at *t* > 0 s. The spikes of the interrupting SB were removed in (A) and (B) for clarity. Successful stimuli evoked responses at *t* > 0 s. Blue lines mark the period of latency prior to the stimulus at *t* = 0 s Magenta triangles indicate stimuli delivered in preceding trials. Within training rounds, the controller was free to explore the state space. Note that these rounds are in closed-loop mode but with a random sequence of stimulation latencies. The strategy in this example was non-greedy. During testing rounds the hitherto best policy was chosen. After the final round, a latency of ≈ 1.4 s was learned. Stimulus properties were as in Fig 1. (B) Zoom-in on responses evoked throughout the session. Interrupted trials appear as empty rows; in this example all stimuli elicited responses. (C) Stimulus efficacy estimated as the response strength per SB (RS/SB) computed over each of the training/testing rounds. RS/SB improved considerably during testing compared to the training rounds. The fraction of trials interrupted in each round is shown as red circles and numerically. The dashed line was added for clarity.

We then proceeded with the closed-loop learning session. The session proceeded in alternating pairs of training and testing rounds. During training rounds, the controller was free to explore the state-action space and update its action-value function estimates, while in a testing round, it always chose an optimal policy based on the knowledge hitherto acquired. The time taken to run through with the experiment varied across networks, but was typically around 3–5 hours, typically covering 1000 SBs. This variability was due to differences in the average burst rate between networks. The latency chosen by the algorithm during the final testing session was considered the learned latency. To test the stability of the learned latency some of the sessions were run with up to 3000 SB in further training and testing rounds. Fig 6 illustrates a typical session in an example network. In this case learning proceeded in three pairs of 200 training and 50 testing trials. Note that a trial in our paradigm refers to the period between SBs where stimulation can potentially be delivered. Each trial is therefore initiated by ongoing activity (SB termination) and not by stimuli. Some of the trials were interrupted by ongoing activity, resulting in stimulus counts less than the planned number.

**Fig 7:**
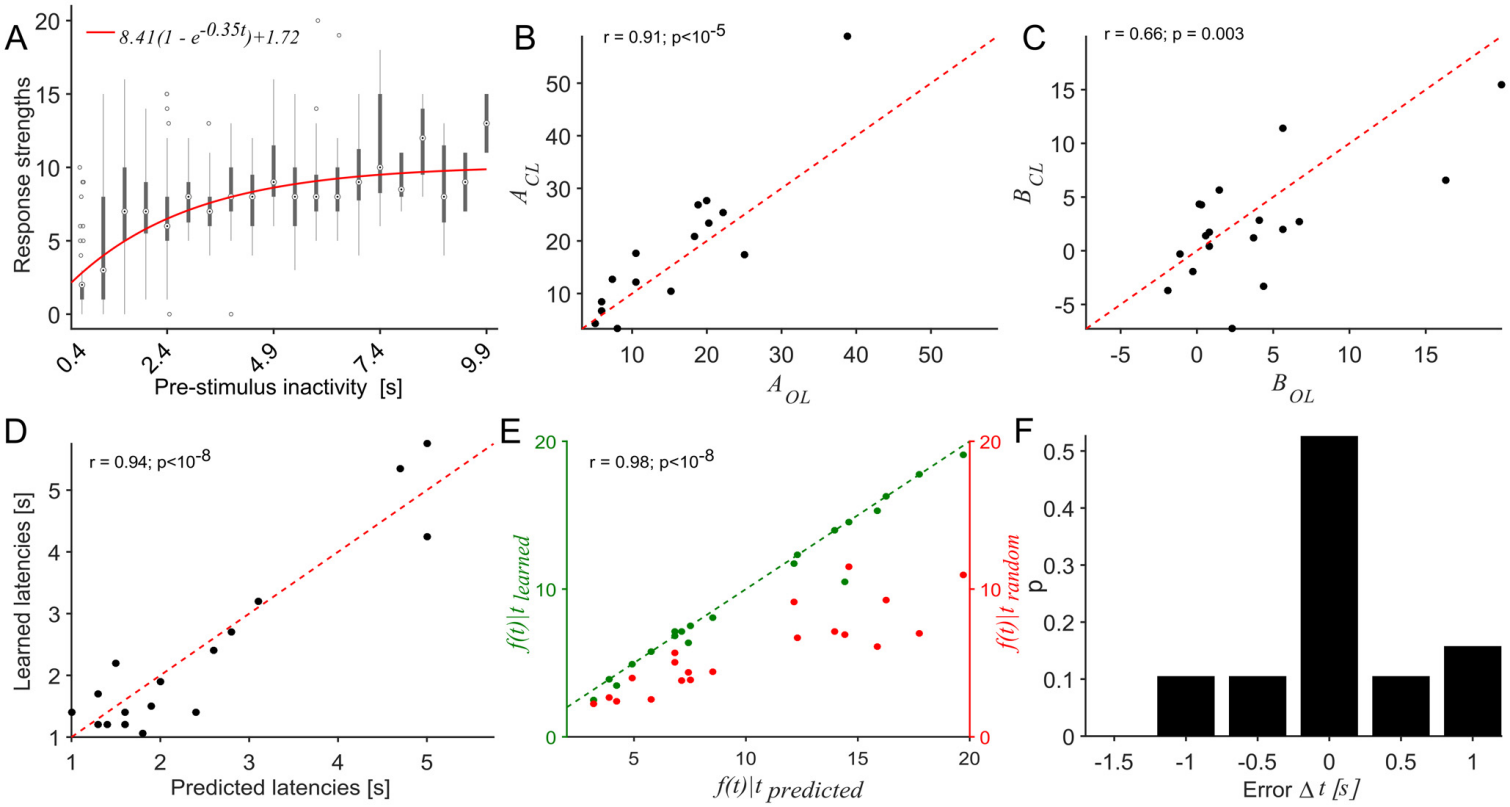
Comparison of open-loop predictions with autonomously learned strategies. (A) Dependence of response strengths on pre-stimulus inactivities in data during a closed-loop session in an example network. Each box shows the statistics of response strengths recorded at one discrete state. The central measures are median and the edges with 25^th^ and 75^th^ percentiles. Whiskers extend to the most extreme data points not considered outliers, and outliers are plotted individually. The fit (red) was made to the medians. The minimal latency for burst termination was 0.4 s in this example, which was thus the earliest state available for stimulation. (B) Across networks, closed-loop estimates of the gain *A* correlated strongly with open-loop estimates (r = 0.91, p<10^-5^, n = 15 networks), indicating that *A* was mostly stable during the experiments. (C) Similarly, closed-loop estimates of *B* were in agreement with open-loop ones (r = 0.66, p = 0.003, n = 18 networks), although to a lesser degree. (D) Across networks, learned stimulus latencies show a positive correlation with predicted optimal values (r = 0.94, p<10^-8^, n = 17 networks). (E) In spite of some variability in Panels B-D the magnitudes of the modeled objective functions for predicted and learned latencies matched closely (green dots), indicating that the network/stimulator system was performing at a near optimal regime, regardless of slight discrepancies in the latencies. Exact optima were likely unreachable owing to the coarse discretization (0.5 s) of states. Red dots denote the corresponding magnitudes at *t*_*rand*_ for a strategy delivering stimuli at random latencies estimated as the mean of the objective function. (F) The distribution of errors between learned and predicted latencies is centered around the predicted optimum and confined to within 2 discrete steps from it.

To analyze the closed-loop sessions, we first looked at the model parameters of the recovery function, *A*, *B* and *λ* and compared values predicted from open-loop sessions with those recovered from fits to the closed-loop data. Note that in this paradigm responses are available only at fixed latencies corresponding to the state definition (Fig 7A). The gain of the network, *A*, showed a strong positive correlation to the open-loop ones (r = 0.91, p <10 ^-5^, n = 15 networks, Fig 7A and 7B), indicating relative stationarity of the quantitative relationship and its accessibility for the controller. Parameter *B*, which can be interpreted as the excitability threshold for SB termination, too showed positive correlation (r = 0.66, p = 0.003, n = 18 networks, Fig 7C), but weaker than model parameter *A*, suggesting that SB termination may depend on additional factors not captured by the model. Parameter *λ* showed a still weaker correlation (S1C Fig). Across networks, closed-loop estimates of the recovery model were thus mostly consistent with open-loop estimates.

We then compared the learned stimulus latencies with those predicted from open-loop sessions. Overall, stimulus latencies learned by the controller showed a strong positive correlation with the optimal latencies estimated from open-loop experiments (r = 0.94, p<10^-8^, n = 17 networks, Fig 7D). Nevertheless, in some networks learned latencies differed from predicted ones, as visible in their distances to the diagonal in Fig 7D. Next, we compared the measure being maximized—stimulation efficacy estimated as the response strength per SB, corresponding to learned and estimated latencies. The network-specific model of the objective function, *f*(*t*) (Eq 6) was used to estimate the maximal stimulation efficacy *f*(*t*)|*t* achievable with the predicted optimal latency vs. the one learned for a given network. Values of this measure were in strong agreement (Fig 7E), indicating that the control goal was achieved despite errors in predicted latencies (Fig 7D). One possible source of errors could be the discretization of the controller’s state space into 0.5 s steps. Indeed, the error distribution showed that 74% of the networks studied fell within ±0.5 s around the optimum (Fig 7F).

**Fig 8:**
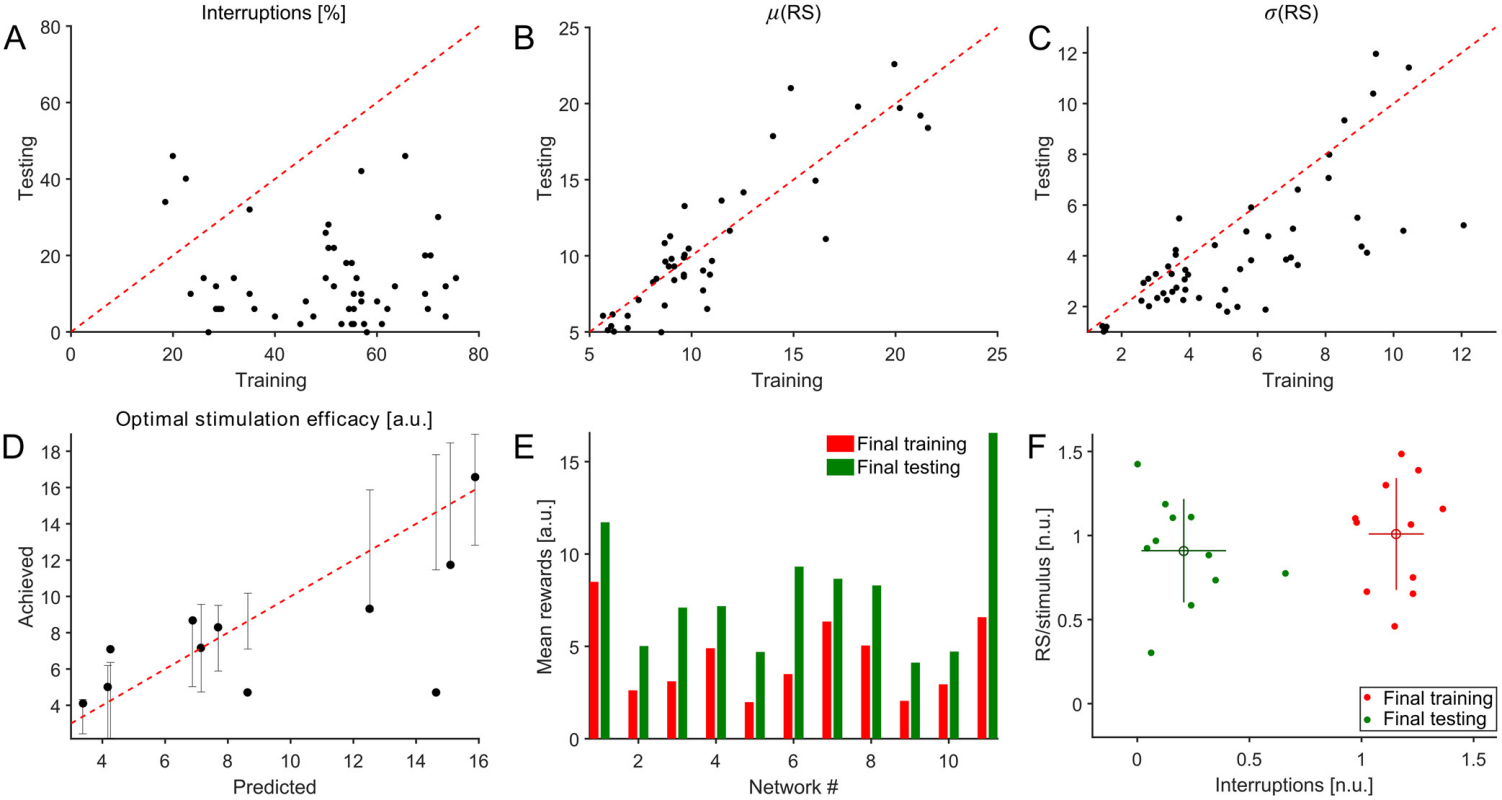
Performance evaluation of the controller. (A) The percentage of interrupted trials during training (x-axis) and testing (y-axis) sessions (n = 52 pairs across 11 networks). This percentage decreased sharply after learning in 94.2% of the recorded sessions. (B) The mean RS evoked per stimulus was, however, preserved in both sessions. (C) The variability in RS per stimulus decreased significantly (p = 0.01, two-sample t-test). (D) Comparison of the optimal stimulus efficacies predicted from our models with the efficacies achieved during the final closed-loop testing sessions. Vertical bars represent 99% confidence intervals corresponding to the models fitted for each network. Achieved values fall within the interval in 8/11 networks studied. (E) Mean rewards were calculated over trials in the final training and testing rounds to compare the controller’s performance. After learning, mean rewards increased in each network, which is indicative of the improvement in stimulation efficacy. The rewards across the sequence of trials in each round were drawn from distinct distributions in every network (p<0.002, two-sample Kolmogorov-Smirnov test). The individual distributions are shown in S2 and S3 Figs. (F) Summary of learning across networks on a normalized RS/stimulus vs. interruption probability plane (11 networks). Only final training and testing rounds were considered. Normalization for interruptions was performed relative to the model-based estimate of interruption probabilities, corresponding to stimulation at random latencies for each network. The RS/stimulus measure was similarly normalized to the model-based estimates of the efficacy assuming a random stimulation strategy. The improvement in performance clearly separates the data points in the plane. Of the two modalities that contribute to stimulus efficacy, the improvement was dominated by reduction of interruption probabilities.

Finally, the performance of the controller was evaluated with respect to the defined goal: to maximize stimulation efficacy measured as the total number of response spikes evoked for every detected SB in the network. A session-by-session analysis showed that in 94.2% of the sessions (n = 52 sessions with non-greedy training, 11 networks), the percentage of interrupted events per session diminished post learning (Fig 8A). IBI distributions of spontaneous activity prior and subsequent to closed-loop sessions showed small changes in only a few networks (p<0.001 in 6/20 networks, two sample Kolmogorov-Smirnov test S4 Fig) and in these, the frequency of IBIs less than 5 s could change in both directions.

While the number of spikes in a response did not significantly change across sessions (Fig 8B) the standard deviation across stimuli in a session decreased (Fig 8C) (p = 0.01, two-sample t-test). Concurrently, in 90% of the cases (n = 52 sessions, 11 networks), the stimulus efficacy had increased after learning, supporting the effectiveness of the learning algorithm.

The models used to estimate the objective functions were derived from fits to spontaneous activity and noisy samples of responses in open-loop experiments. The quality of the predictions thus depended on the reliability of these fits. Comparison of the optimal stimulus efficacies predicted from our models with the efficacies achieved during the final closed-loop testing sessions showed that achieved efficacies were within the 99% confidence interval for the models fitted for each network (Fig 8D). Achieved stimulus efficacies fall within the interval in 8 of 11 networks studied.

Learning clearly improved performance in each network (p<0.001, two-sample Kolmogorov-Smirnov test). The amount of improvement, however, varied across networks (Fig 8E). To compare performance across networks, we captured each network on a normalized response-per-stimulus vs. interruption probability plane (Fig 8F). Each network is shown before and after learning. Only the last pairs of sessions were used for this plot (n = 11 networks). The distribution shows a clear separation of the mixed-mode performance before and after learning, indicating the improvement of stimulation efficacy. The improvement was almost exclusively due to a reduction in interruption probability (S2 and S3 Figs). This, however, also says that the controller learns to avoid losing in response magnitude by not further reducing the interruption probability, i.e. it balances the trade-off.

## Discussion

Closed-loop stimulation has been proposed as a promising strategy to intervene in the dynamics of pathological networks while adapting to ongoing activity. The selection of signal features to close such a loop and strategies to identify optimal stimulus settings given a desired network response, remain open problems. We propose methods of reinforcement learning to autonomously choose optimal control policies given a pre-defined goal. In this study, we present proof of principle of such a controller interacting in a goal directed manner with generic neuronal networks *in vitro*, boundary conditions for such interactions and an analysis of the optimality of the learned controller.

Our results demonstrate the capacity of RL based techniques to autonomously exploit quantitative relationships underlying a complex network of neurons to find optimal actions. Drawing on previous studies, we identified a simple verifiable trade-off scenario in which the dynamics of spontaneous activity in these networks interact with external electrical stimuli [20]. The temporal relationship of SB events in these networks may be approximated by a lognormal function. Moreover, response strengths to electrical stimuli have been shown to quantitatively fit a saturating exponential model, dependent on the stimulus latency subsequent to an SB event. Interaction of these underlying processes gives rise to an abstract objective function predicting a network specific and unique stimulus latency relative to a SB that maximizes the number of response spikes evoked per SB across repeated stimulation. The goal set for the RL controller was to autonomously identify this optimal stimulus latency.

Response strength was defined as the number of spikes detected in a predefined temporal window (typically 500 ms) from stimulus onset. Note that while [20] define response lengths as the time to the last spike of the detected response, our data showed that the spike counts in a temporal window post stimulus is proportional to the response lengths measured in time and hence can be used as an alternative variable.

This toy problem for the model system captures some of the major challenges that closed-loop paradigms would face in a biomedical application, i.e. in a very complex, adaptive environment. Balancing the trade-off of response magnitude and interruptions involves finding the dependence of response magnitudes on stimulus latencies and adjusting at the same time to the distribution of ongoing activity. With every network being distinct in the properties of its spontaneous dynamics and response to stimuli, the paradigm was tested for robust operation over a range of parameters. Furthermore, the ongoing activity is highly variable and subject to unpredictable modulation at a wide range of time scales. Plasticity of synaptic coupling could lead to further challenges. Prior studies on such networks enable us to model this interplay using parametric models under the assumption of system stationarity. This provided us with the convenient situation where an open-loop prediction of the network-specific optimal stimulus latency could be calculated to evaluate the quality of the controller’s learned strategy. Furthermore, both main processes involved could be modeled as a function of only the latency following an SB event, which thus became an informative and quantifiable low-dimensional state feature for the RL controller. Other contributing factors could have been the recent history of spontaneous activity. Offline analyses, however, did not show an influence of this on the quality of the response properties.

We used numerical models of the system to identify the interactions of variations in the model parameters. The numerical approach revealed the multi-modal nature of the control problem—in that two separate measurable modalities are simultaneously involved– the exponential recovery function and the statistical model of ongoing event occurrence. Combining them enabled us to visualize the non-linear quasi convex input–output dependence *f*(*t*). The desired metric *f*(t*), was unique, distinct for each network and necessarily attainable, because the corresponding t* always belonged to the domain of interest (0–10 s) throughout the span of parameter values observed experimentally. Each network, being a static parameter combination, would therefore map to a single non-linear input–output curve that belonged to the set of objective functions described earlier. In other words, an optimal solution was possible for all parameter sets within the observed range. Open-loop estimates of the saturating exponential model parameters *A* and *B* correlated positively with fits to data from closed-loop sessions. This indicates that the quantitative relationship is stable and accessible to the controller during the learning phase. Note that the temporal stability of the model is a precondition to the controller being able to converge to the same optimal stimulus latencies as the open-loop estimates. Indeed we observed across networks, that the learned optimal stimulus latencies agreed with those predicted from open-loop studies. The controller apparently robustly and autonomously exploited the underlying stimulus-response relationships in the network by interacting with it and adapting appropriately to ongoing activity dynamics.

In this study, stationarity of system dynamics was a necessary assumption in order to be able to compare optimal stimulus latencies predicted from open-loop data at one point in time, to those learned later in closed-loop sessions. However, this might not necessarily be the case. Neuronal networks *in vitro* are known to undergo activity dependent plastic changes [29]. The model parameters of the exponential recovery function are likely time dependent as well. Differences in the magnitudes of correlations between parameters *A* (strongly correlated), *B* (less positively correlated) and *λ*(no correlation) in open vs. closed-loop data fits are possible indicators that some parameters are perhaps more strongly modulated over time than others. For slow fluctuations, the RL paradigm could easily be modified to update the controller, provided the temporal resolution of the state-space is high enough to sample the impact of fluctuations and that the update intervals are adjusted accordingly. It would, however, not be possible to monitor these modulations of network properties during testing in the current paradigm. We would therefore not be able to validate the optimality of resulting controller.

In spite of such sources of variability, the correlation of the learned latencies of the controller with the preceding, temporally distant, open-loop predictions is strong. One reason for this could be the resolution of the controller—the state-space discretization chosen for the controller was relatively coarse at 0.5 s. From our parametric model of the trade-off situation (Figs 4 and 5), it can be seen that the impact of parameter fluctuations on optimal latencies would be small relative to this resolution of the state-space. The actual optimum, thus, could fall in the neighborhood of the learned latencies. Such a tendency is indeed visible in the error between the learned and optimal times (Fig 7F), which are centered around the optimum. Therefore, by coarse graining the state-space discretization we compensate to some degree for non-stationarities in the system. An additional factor contributing to the strong correlation could be rapid parameter fluctuations, which could average out within the duration of the experiment.

In all networks the achieved stimulation efficacy increased after learning, but individually could be below or above the predicted levels Fig 8D. This was probably due to the quality of fits of the recovery function and to non-stationarities in network activity that built up between the time of the original estimate of the objective function and the training/testing sessions eventually available for evaluation. Further contributions to variability could come from the choice of the range of latencies available to the controller for exploration. Longer latencies would lead to increasing probabilities for interruptions during training, biasing the relative success of the learned controller. In this study we set maximal latencies to 10 s, which ensured that recovery would saturate in most networks. Moreover, as Figs 5B and S1A illustrate, the time-constants of these functions also influence the achieved gain in stimulus efficacies.

Although our control goal was drawn on previous studies on the model system, this insight was not implemented into the controller and was used only to validate its performance. However, it must be conceded that our understanding of the underlying relationship did inform the definition of the low-dimensional state-space for the controller, i.e. that the delay of the stimulus to the preceding burst is relevant for the magnitude of the response. This on the one hand was essential to validate the controller’s quality but also reduced the number of trials needed for the controller to converge. In turn, our model cannot capture processes depending on the serial structure of the stimulation sequence, e.g. the rate of stimulation, activity dependent plasticity or even damage to neurons.

Our choice of Q-learning with a tabular representation of the Q-function was motivated as follows. For one, Q-learning allows us to learn a Q-function without having a model of the system dynamics, which in general is not available when dealing with biological systems. Secondly, since the state space for the control task at hand could be defined as a single discrete variable, a tabular representation of the Q-function was applicable, which guarantees convergence [28] (we note that convergence can also be achieved in some cases where the Q-function is approximated, see e.g. Szepesvári [30] for an overview). A tabular representation of the Q-function is a suitable choice as long as the biological system can be described by low-dimensional discretized states. If a different control problem or biological system demands a moderately finer discretization of the state space, tabular Q-learning could still be applied. However, if the control problem requires a state-action space that is high-dimensional or continuous, a tabular representation of the Q-function is not advisable due to the so called curse of dimensionality (exponentially growing memory demand). For our long-term goal of general control of neuronal networks using RL controllers this would clearly be the case.

To this end, future work should further investigate the possibility of feature based approximate RL using e.g. artificial neural networks (ANNs) [31, 32] or Random Forests [33, 34]. As features, such approaches could utilize statistics summarizing network activity in terms of previous burst and response characteristics, adapt features learned from offline data sets from multiple networks [34], or use a machine learning approach to automatically find descriptive features.

In this proof-of-principle study, we show that an RL based autonomous strategy can find optimal stimulation settings in the context of a dynamic neuronal system. Extending on special applications, in which the aim of stimulation is to abolish some type of event, such as epileptic events or oscillations in Parkinson’s Disease, the system studied here represents a more general situation in that the optimal response is initially numerically undefined, i.e. it can take different values depending on the properties of a network that are unknown to the experimenter. It also extends beyond the response clamp paradigm [21] in that it takes into account not only the probability to induce a response but both its magnitude and the probability of being interrupted by spontaneous activity. Our paradigm is thus related to the idea of inducing desired network activity, e.g. towards sensory feedback by stimulation from neuroprosthetic devices, or adjusting the activity dynamics of a network to a desired working mode. In the context of our study, we focused on this specific multi-modal trade-off problem to maximize a derived feature of the response (response strength per SB event). We could show that a unique optimal strategy exists for each network and thus verify that the controller autonomously found the optimum of the objective function given the limitations of our data. Where RL paradigms are applied to more general situations, phenomenological models of their interaction with biological neuronal networks could nonetheless help to estimate the quality of the controllers when full mechanistic descriptions of the system are not available.

## Supporting Information

S1 Fig Dependence of the optimal stimulation latency on the slope of the recovery function and the location and scale parameters of the IBI distributions. (A) t* depends on the shape of the recovery function. t* shifts to later times with increasing recovery slope (*λ* increases) when average inter-burst intervals *μ* are short, i.e. spontaneous activity is high and the probability for interruption is high. In low activity regimes, however, the probability of interruption is low, hence t* is late and increasing the slope will lead to a decrease of the stimulus efficacy with increasing latencies since increasing interruption probability then outweighs the gain in spikes/stimulus. Because of the saturation of recovery changes in the probability for interruptions have a dominating influence on t*. (*inset*) t* shifts to later latencies with increasing *μ* for a given *λ* (boxed). *A*, *B* and *σ* were set to 20, 6.67, 1 respectively. (B) Scale parameter, *σ* of the IBI distribution had little impact on the optimal stimulation latency. *A*, *B* and *λ* and *μ* were set to 20, 6.67, 1 and 0.6 respectively. (C) Across networks, values of *λ* recovered from fits to closed-loop data were uncorrelated with open-loop estimates.
(TIF)

S2 Fig Reward probability distributions for all networks. In each training trial the controller received a reward according to the number of spikes elicited by the stimulus. In trials interrupted by SBs this resulted in neutral reward (−10^−3^), pooled with trials eliciting 0 spikes in the histograms. After learning, the probability for very high rewards was reduced but this was outweighed by the lower frequency of 0 and neutral rewards.
(TIF)

S3 Fig Empirical cumulative distribution of rewards for all networks. The Empirical cumulative distribution function (ECDF) of the rewards clearly shows that the improvements by learning were dominated by reduced probabilities to receive 0 or neutral rewards.
(TIF)

S4 Fig Distributions of IBIs before and after closed-loop sessions. Distributions of IBIs in spontaneous activity recorded before (blue) and after (yellow) closed-loop sessions. A two-sample Kolmogorov-Smirnov test showed that the IBIs were drawn from distinct distributions in 6/20 networks (p<0.001, bold axes).
(TIF)
