# Supplementary figures and images for: Autonomous Optimization of Targeted Stimulation of Neuronal Networks
Source: PLoS Comput Biol. 2016 Aug 10;12(8):e1005054. doi: 10.1371/journal.pcbi.1005054 (PMC4979901; doi:10.1371/journal.pcbi.1005054)

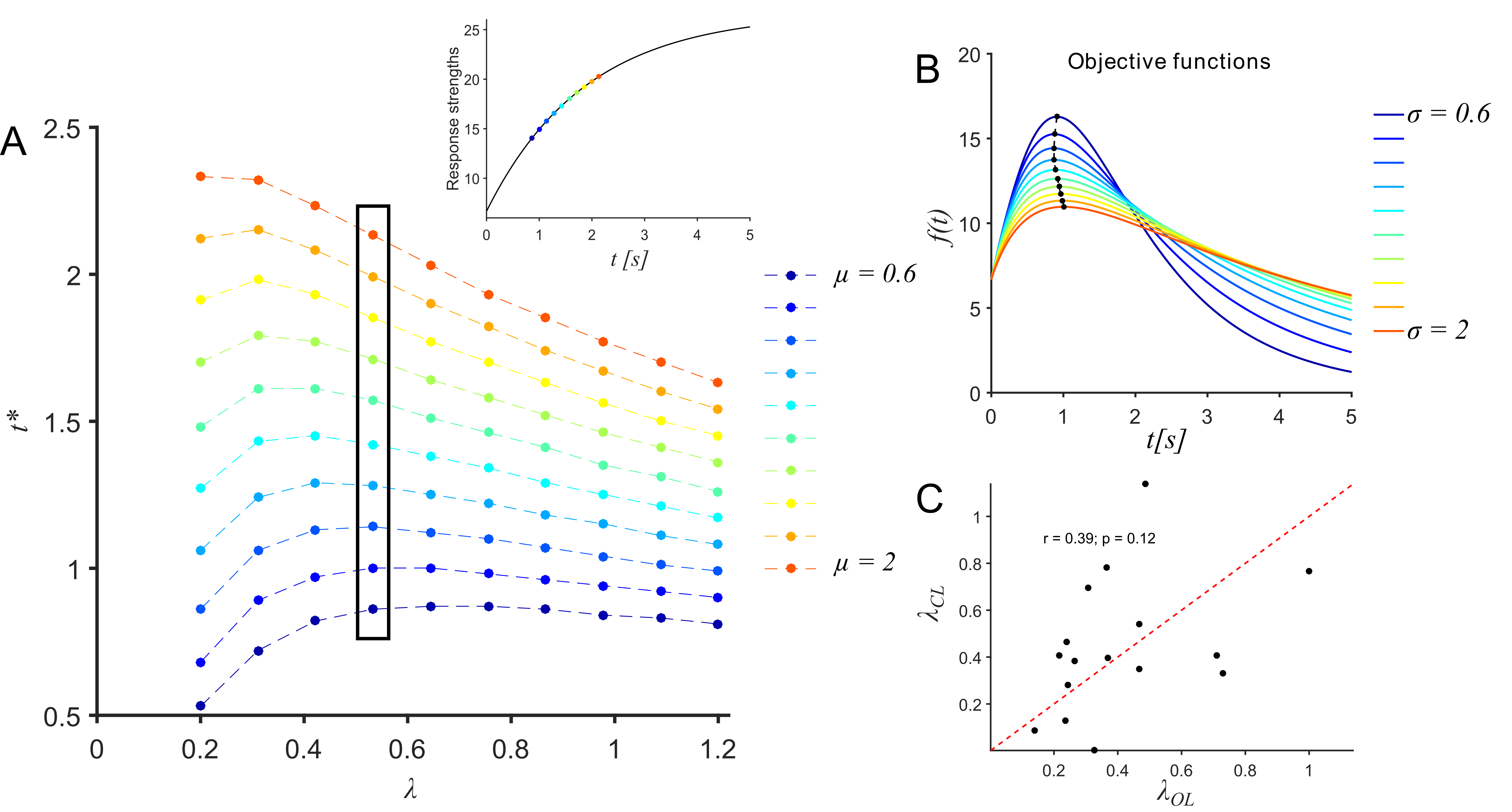

Supplement: S1 Fig — (A) t* depends on the shape of the recovery function. t* shifts to later times with increasing recovery slope (λ increases) when average inter-burst intervals μ are short, i.e. spontaneous activity is high and the probability for interruption is high. In low activity regimes, however, the probability of interruption is low, hence t* is late and increasing the slope will lead to a decrease of the stimulus efficacy with increasing latencies since increasing interruption probability then outweighs the gain in spikes/stimulus. Because of the saturation of recovery changes in the probability for interruptions have a dominating influence on t*. (inset) t* shifts to later latencies with increasing μ for a given λ (boxed). A, B and σ were set to 20, 6.67, 1 respectively. (B) Scale parameter, σ of the IBI distribution had little impact on the optimal stimulation latency. A, B and λ and μ were set to 20, 6.67, 1 and 0.6 respectively. (C) Across networks, values of λ recovered from fits to closed-loop data were uncorrelated with open-loop estimates. (TIF) [file pcbi.1005054.s001.tif]

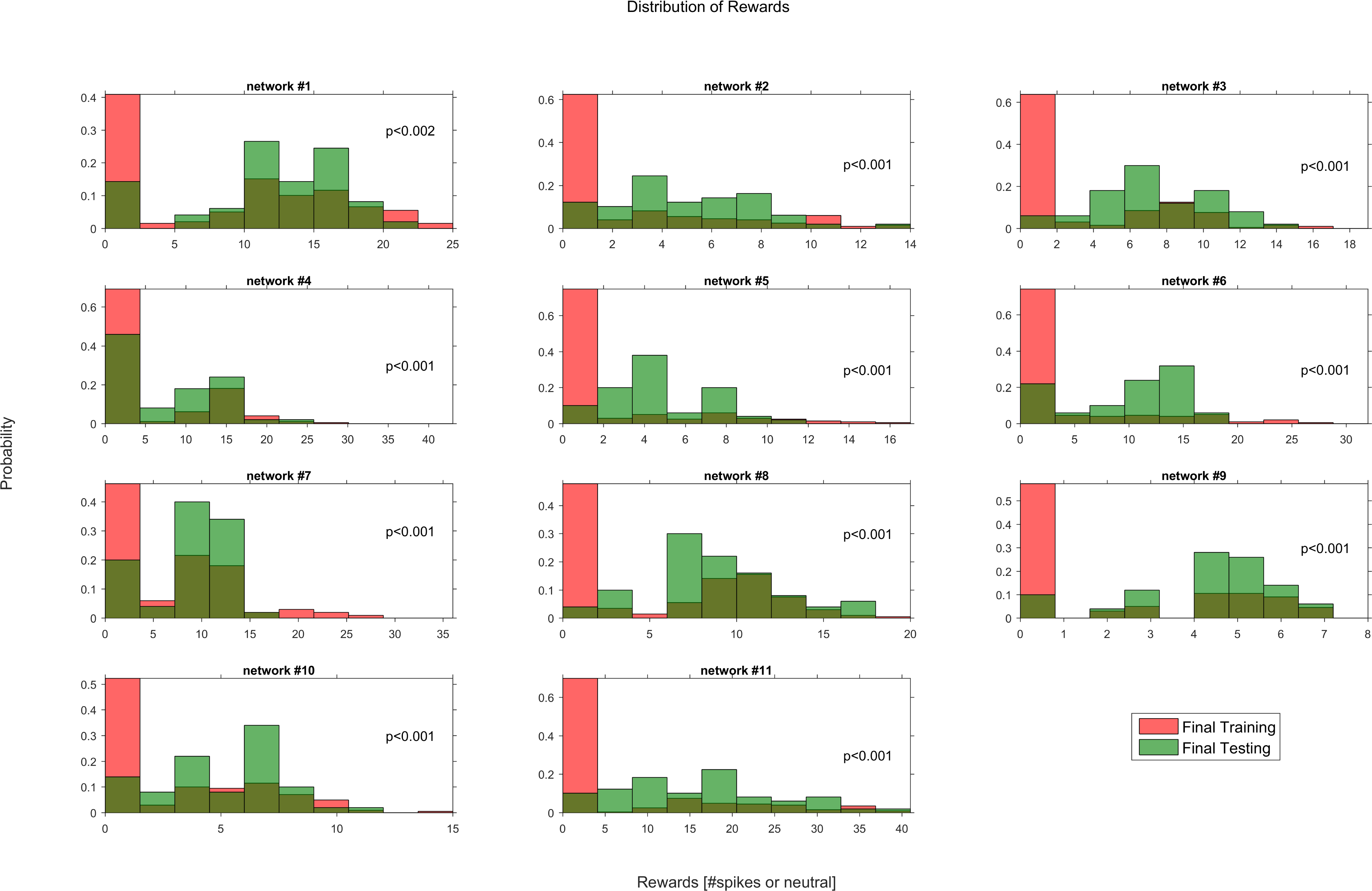

Supplement: S2 Fig — In each training trial the controller received a reward according to the number of spikes elicited by the stimulus. In trials interrupted by SBs this resulted in neutral reward (−10−3), pooled with trials eliciting 0 spikes in the histograms. After learning, the probability for very high rewards was reduced but this was outweighed by the lower frequency of 0 and neutral rewards. (TIF) [file pcbi.1005054.s002.tif]

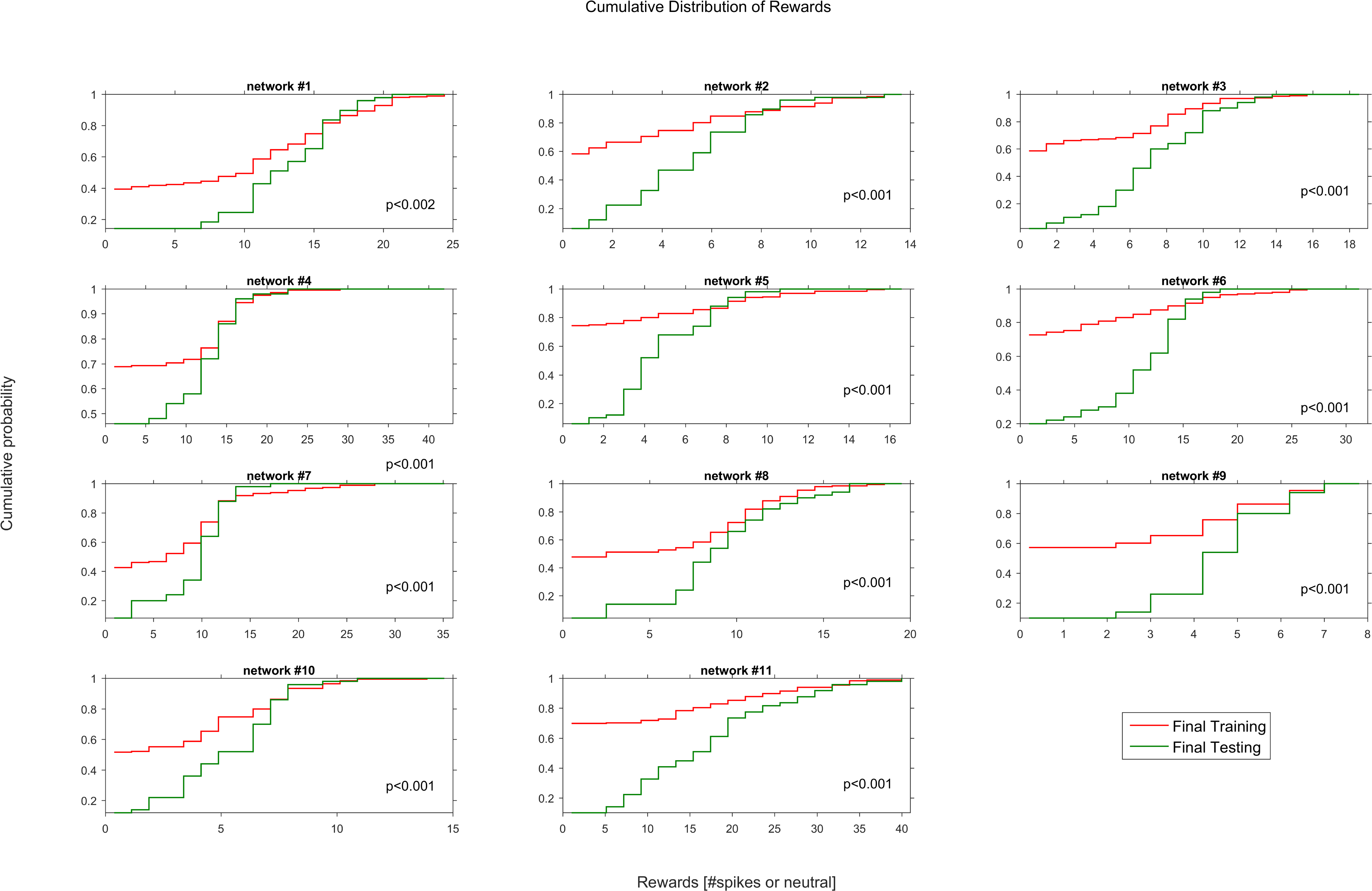

Supplement: S3 Fig — The Empirical cumulative distribution function (ECDF) of the rewards clearly shows that the improvements by learning were dominated by reduced probabilities to receive 0 or neutral rewards. (TIF) [file pcbi.1005054.s003.tif]

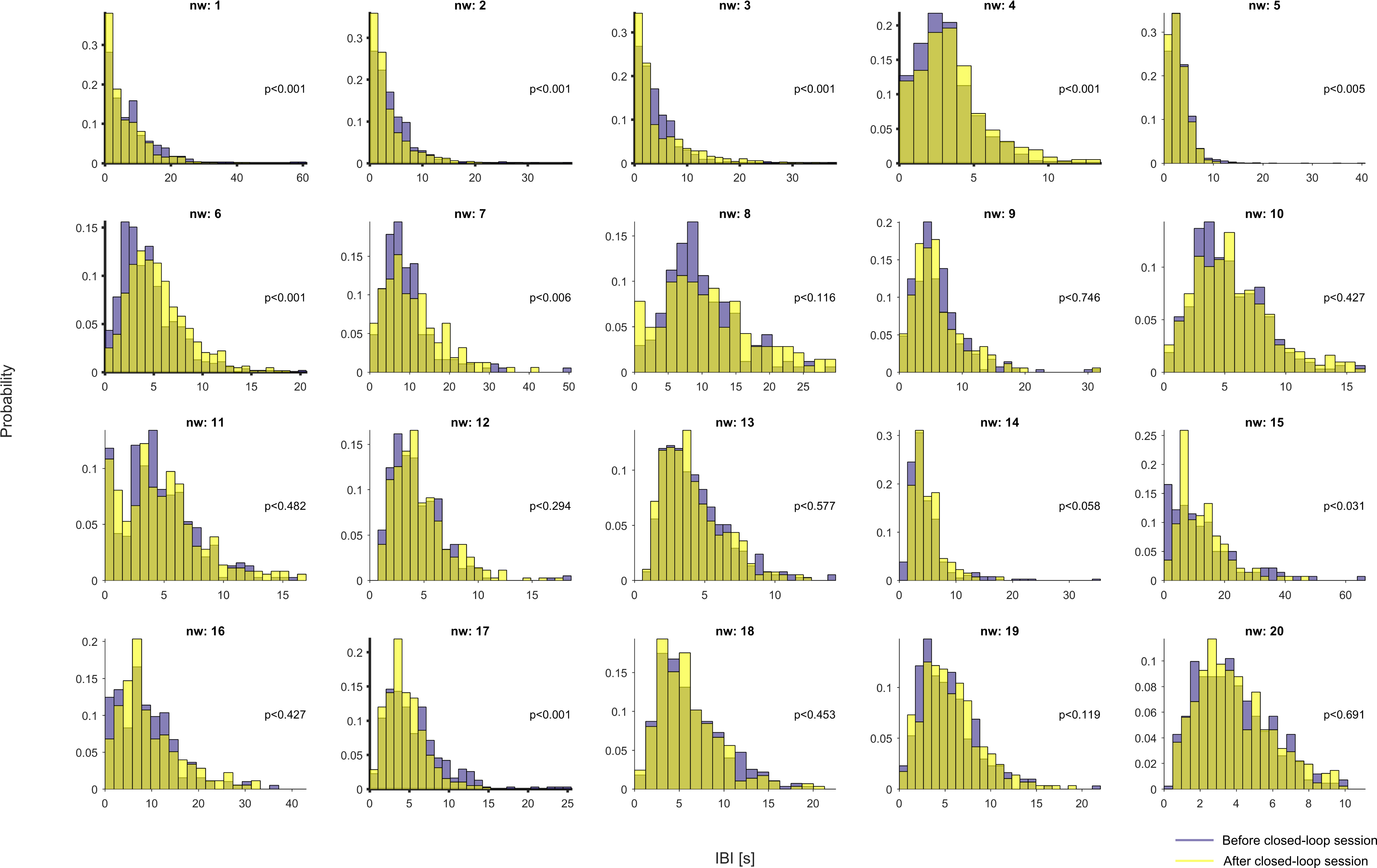

Supplement: S4 Fig — Distributions of IBIs in spontaneous activity recorded before (blue) and after (yellow) closed-loop sessions. A two-sample Kolmogorov-Smirnov test showed that the IBIs were drawn from distinct distributions in 6/20 networks (p<0.001, bold axes). (TIF) [file pcbi.1005054.s004.tif]
